# The current state of the use of DNA markers for improving the efficiency of rice breeding in Japan

**DOI:** 10.1270/jsbbs.25060

**Published:** 2026-02-20

**Authors:** Akitoshi Goto, Kei Matsushita, Utako Yamanouchi

**Affiliations:** 1 Institute of Crop Science, NARO, 2-1-2 Kannondai, Tsukuba, Ibaraki 305-8518, Japan

**Keywords:** rice, gene, QTLs, marker-assisted selection, breeding efficiency

## Abstract

In response to recent environmental and geopolitical changes, Japan requires rice cultivars with high adaptability and production efficiency. Advances in rice genomics have enabled precise genetic mapping and the use of DNA markers for efficient selection. Marker-assisted selection (MAS) reduces labor and environmental influences, and co-dominant markers allow accurate genotyping. When combined with backcrossing, MAS enables rapid pyramiding of multiple traits. Marker-assisted breeding has been widely applied to improving elite cultivars across Japan. This review highlights the practical applications of DNA markers in rice breeding programs in Japan. Specifically, we examine how molecular markers have been used to improve resistance to diseases and pests, improve grain quality, strengthen tolerance to abiotic stresses, and support the selection of agronomically important quantitative traits such as heading date and yield. We also provide an overview of a practical approach to accelerating breeding through MAS and generation advancement for efficient trait stacking. Finally, we present future perspectives on expanding the use of molecular markers to further improve the efficiency and precision of rice breeding.

## Introduction

Japan consists of a long, narrow, bow-shaped group of islands stretching from northeast to southwest, with a diverse range of growing environments. Rice (*Oryza sativa* L.) breeders have selected many excellent cultivars that are suited to the environment of each region, supporting the production of rice as a staple food. However, global warming has accelerated in recent decades (IPCC 2021), requiring the implementation of measures to cope with the increasing incidence of newly emerging diseases and heat stress, as well as shifts in the maturity timing of individual cultivars. In addition, geopolitical instability has had a profound impact on the global fertilizer market, leading to a significant increase in the production costs of major crops (USDA 2022). It is therefore necessary to rapidly develop elite cultivars that are adapted to the environment of each region and can be produced sustainably and at low cost.

To efficiently develop new rice cultivars, it is essential to accurately understand the genome structure associated with desirable traits. Along with the establishment of rice genomics through projects such as the International Rice Genome Sequencing Project (Matsumoto *et al.* 2005), quantitative trait locus (QTL) analysis has greatly enhanced our understanding of natural variation in rice, providing extensive information on the chromosomal locations and allelic effects of gene loci that are valuable for breeding programs (Yamamoto *et al.* 2009). The analysis of next-generation sequencing (NGS) data, supported by developments in bioinformatics, has further contributed to the identification and characterization of novel genes and regulatory sequences, including their genomic positions, thereby expanding the availability of DNA markers for genetic research and breeding applications (Pérez-de-Castro *et al.* 2012). The widespread availability of DNA markers has promoted the application of advanced genetic analysis methods, such as genome-wide association studies (GWAS). GWAS offer advantages over traditional bi-parental mapping by accounting for allelic diversity and various genetic backgrounds across the entire accession collection within a breeding program (Adjah *et al.* 2022). This approach has significantly accelerated the identification of breeding-relevant QTLs across a wide range of populations analyzed. Consequently, the genetic maps used for cultivar improvement are becoming increasingly precise and informative.

Marker-assisted selection (MAS) is an indirect selection process that utilizes polymorphisms in DNA markers associated with desirable traits and serves as an effective strategy for enhancing breeding efficiency. Compared to conventional selection methods based on phenotypic screening, MAS can reduce the time, resources, and labor required for breeding programs (Collard and Mackill 2008). For example, conventional screening for disease and insect resistance in rice often requires dedicated space for insect rearing or fungal culturing and inoculation, along with a significant amount of labor. Moreover, environmental conditions and human error frequently lead to ambiguous evaluation results (Fujii *et al.* 2023, Makkar *et al.* 2019, Srinivasachary *et al.* 2002). In contrast, MAS enables the identification of desirable individuals for any trait, or combination of traits, as early as the seedling stage. This allows breeders to determine whether promising breeding materials possess useful traits even in the early generations, before they are developed into stable breeding lines (Collard and Mackill 2008). Moreover, because selection from segregating population is based on genetic information, the results are not influenced by environmental conditions. When selection is based on phenotypic values, it is often difficult to distinguish whether plants are homozygous or heterozygous for the target QTL (Fujino *et al.* 2019). On the other hand, when using co-dominant DNA markers such as simple sequence repeats (SSRs) or single-nucleotide polymorphisms (SNPs), it is possible to determine whether a gene is homozygous or heterozygous in individual plants, which facilitates more precise decision-making in the management of breeding materials (Collard and Mackill 2008).

Most elite cultivars that are widely grown across various regions exhibit no major issues in their agronomic traits, but may have shortcomings. To improve the shortcomings of such elite cultivars, marker-assisted backcrossing (MABC), which combines MAS with backcrossing, is an effective approach. MABC is the process of using markers to select for target loci, minimize the length of the donor chromosomal segment containing a target locus and/or accelerate the recovery of the recurrent parents genome during backcrossing (Hospital 2001, Hospital and Charcosset 1997). It takes a minimum of 6–8 backcrosses to fully recover a recurrent parent genome using conventional breeding methods based on phenotypic surveys, but MABC enables the procedure to be shortened to 3 or 4 backcrosses (Tanksley *et al.* 1989). It typically takes about 10 years to develop and release a new rice cultivar through conventional breeding programs, but the use of MABC can significantly shorten the process. For example, in a case where strong blast resistance was introduced into a local elite cultivar, the breeding period was successfully reduced by almost half, to six years, even when including adaptability testing in the target cultivation region (Matsumoto *et al.* 2023).

In crop breeding, the development of durable resistance is considered essential, as both pathogens and insect pests frequently overcome single-gene host resistance over time owing to the emergence of new pathogen races and insect biotypes (Hasan *et al.* 2015). Some evidence suggests that the combination of multiple genes, each effective against one or more specific races of pathogens or biotypes of insect pests, can provide durable and broad-spectrum resistance (Hu *et al.* 2013, Qiu *et al.* 2012, Shanti *et al.* 2001, Sharma *et al.* 2004, Singh *et al.* 2001). In conventional phenotypic evaluations, it is difficult to accurately determine the types of resistance genes introduced into a plant, and the process requires considerable time and effort (Hasan *et al.* 2015). In contrast, MAS enables the rapid and reliable identification of the presence or absence of target genes. Moreover, the pyramiding of desirable resistance genes through MABC allows for the efficient development of disease- and pest-resistant cultivars with significantly reduced labor. When attempting to improve multiple traits simultaneously, conventional methods require separate phenotypic evaluations for each trait, whereas MAS enables selection of multiple traits at once within a single laboratory setting.

Marker-assisted breeding using MAS and MABC has been widely adopted as an efficient strategy to accelerate breeding, with many practical applications reported across diverse breeding programs worldwide (Adjah *et al.* 2022, Agarwal *et al.* 2016, Jena and Mackill 2008, Khanh *et al.* 2021, Rao *et al.* 2014). In Japan, marker-assisted breeding began with efforts to improve the drawbacks of ‘Koshihikari’, the most widely cultivated and commercially popular rice cultivar. By using marker-assisted breeding, ‘Koshihikari Aichi SBL’ (Sugiura *et al.* 2004), which carries resistance to stripe disease and panicle blast—two major weaknesses of ‘Koshihikari’—was developed and submitted for cultivar registration in 2002. Subsequently, ‘Koshihikari Tsukuba SD1’, which features improved lodging resistance, was submitted for registration in 2004 (Wang *et al.* 2005). Building upon this foundation marker-assisted breeding has been widely applied to the improvement of many elite local cultivars across Japan, significantly contributing to the overall enhancement of varietal performance nationwide. Table 1 presents the genes and QTLs that have been utilized in the development of registered cultivars within Japanese breeding programs. Other genes and QTLs expected to be useful for breeding new cultivars are listed in Supplemental Table 1. Each of these will be described in detail in the subsequent sections of this review.

In this review, we highlight the practical applications of DNA markers in rice breeding programs in Japan. Specifically, we examine how molecular markers have been utilized to improve resistance to diseases and pests, improve grain quality, strengthen tolerance to abiotic stresses, and support the selection of agronomically important quantitative traits such as heading date and yield. We also provide an overview of a practical approach to accelerating breeding through MAS and generation advancement for efficient trait stacking. Finally, we present future perspectives on expanding the use of molecular markers to further enhance the efficiency and precision of rice breeding.

## Application of DNA markers for improvement of disease and pest resistance

### Improvement of resistance to rice blast

Rice blast caused by the fungal pathogen *Magnaporthe oryzae* is the most devastating disease in rice cultivation in Japan. Use of cultivars that carry resistance to this disease is a cost-effective and environmentally friendly means to control the pathogen (Kitazawa *et al.* 2019). Resistance to rice blast is generally categorized into two types: true resistance and field resistance. True resistance is qualitative and cultivar-specific, typically governed by single major resistance genes that confer strong but race-specific protection. In contrast, field resistance is non-race-specific and is controlled by multiple QTLs, providing partial resistance to many races.

Genotypes with true resistance for blast are conventionally identified by inoculating with differential pathogen strains to test sample rice cultivars against reference cultivars for respective resistance alleles (Hayashi 2015). Such conventional systems used in the Japanese rice breeding program have discriminated 12 resistance alleles (*Pik-s*, *Pia*, *Pii*, *Pik*, *Pik-m*, *Piz*, *Pita*, *Pita-2*, *Piz-t*, *Pik-p*, *Pib*, and *Pit*) (Kiyosawa 1984). In recent years, many high-yielding cultivars for animal feed have been bred with an *indica* genetic background, leading to increased diversity in allelic combinations for blast resistance (Kato 2008, Yonemaru *et al.* 2014). To address the increased diversity, a novel differential system was proposed (Hayashi and Fukuta 2009); however, this method requires a greater number of differential isolates and reference cultivars than conventional approaches, and thus demands significantly more labor and expertise.

Genetic mapping of genes for blast resistance has contributed to the development of DNA markers for resistance alleles (Hayashi *et al.* 2004, 2006, 2010a, Koide *et al.* 2009, Nonoue *et al.* 2018, Tian *et al.* 2016, Wu *et al.* 2015), which have been used to efficiently introduce resistance in rice breeding programs (Ashkani *et al.* 2015, Hasan *et al.* 2015). Kitazawa *et al.* (2019) developed a high-precision genotyping system using the Fluidigm SNP genotyping platform, constructing a set of 96 SNP markers for 10 loci controlling race-specific resistance. This system enables identification of the presence or absence of 24 resistance alleles with a classification accuracy of 93.5%, which is consistent with genotypes determined by inoculation tests in *japonica* cultivars. Furthermore, it has been successfully applied to evaluate resistance alleles that were previously difficult to distinguish using inoculation-based methods. At present, the genotyping data for true blast resistance obtained using this marker set are considered highly reliable, and their use has been officially approved in the national cultivar registration process conducted by the Japanese government (MAFF 2024).

Although true resistance is highly effective against blast, its durability is limited owing to the potential for resistance breakdown, even when multiple true resistance genes are pyramided into a single cultivar. ‘Tongil’ is a high-yielding rice cultivar developed in South Korea through inter-subspecific crossing between *indica* and *japonica* rice. Although it carries multiple true resistance genes including *Pia* and *Pib*, its resistance to blast broke down within six years of widespread cultivation, leading to its eventual decline in use (Kiyosawa and Cho 1980). ‘Hama-asahi’, a rice cultivar developed in Aomori Prefecture by pyramiding four major blast resistance genes (*Pia*, *Pii*, *Pik*, and *Pib*) was also reported to have experienced a breakdown of resistance (Kiyosawa *et al.* 1984). Therefore, instead of pyramiding multiple major resistance genes into a single cultivar, efforts have been made to enhance resistance to rice blast through the development and use of multiline varieties, which are composed of lines that are genetically uniform lines for agronomic traits but that each carry a different major resistance gene. In Japan, this approach has been practically implemented through the development of multiline varieties of elite cultivars such as ‘Sasanishiki’, ‘Koshihikari’, and ‘Hanaechizen’, which have been effectively deployed to manage blast outbreaks in the field (Abe 2004, Ishizaki *et al.* 2005, Tomita *et al.* 2006).

The widespread adoption of DNA markers has improved the efficiency of breeding and seed management in multiline varieties by enabling clear identification of the presence or absence of specific genes used in these lines (Nakamura *et al.* 2006, Tomita *et al.* 2006). Many multiline varieties are composed of lines carrying major resistance genes that were previously introduced but have since lost their effectiveness owing to pathogen adaptation, so additional genes are continually required. The *Pi9* gene (Qu *et al.* 2006), derived from the wild rice species *Oryza minuta*, confers broad-spectrum resistance, exhibiting strong or moderate resistance to 31 isolates collected from Asia and Africa in blast bioassays (Fukuta *et al.* 2022). ‘Koshihikari Kanto BL1’, a cultivar in which the *Pi9* gene has been introduced into the genetic background of ‘Koshihikari’ through MABC, has been developed (Tsunematsu *et al.* 2015). This genetic material is considered useful for improving the composition of multiline varieties in Japan.

True resistance to rice blast is typically associated with hypersensitive response and localized cell death. However, among field resistance genes that do not trigger such responses, there are cases where a single gene can confer strong resistance. *Pb1* is a field resistance gene derived from the *indica* rice cultivar ‘Modan’ of Pakistani origin, which confers strong resistance to panicle blast and whose expression increases with plant maturity (Hayashi *et al.* 2010b). No breakdown of resistance has been reported for over 40 years since the dissemination of *Pb1*-containing cultivars, making them a durable and reliable genetic resource for enhancing blast resistance in rice (Fujii *et al.* 2023). Although cultivars such as ‘Satojiman’ and ‘Hoshijirushi’ carry *Pb1*, they exhibit limited resistance to panicle blast. This reduced effectiveness is not due to the breakdown of *Pb1*-mediated resistance by pathogen evolution, but rather is attributed to the presence of four QTLs that impair salicylic acid signaling, which is essential for the activation of *Pb1*-dependent resistance (Inoue *et al.* 2017). The effective utilization of *Pb1* for panicle blast resistance can be stably achieved by incorporating a set of expression-regulating QTLs that ensure its strong and consistent expression. *Pb1* has been introduced into many recent Japanese cultivars through MAS. Moreover, its application in blast resistance breeding has also been reported outside Japan, including in countries such as South Korea and Bangladesh, where it has been used in combination with DNA markers to develop resistant lines (Lee *et al.* 2015, Nihad *et al.* 2024).

**Fig. 1.**
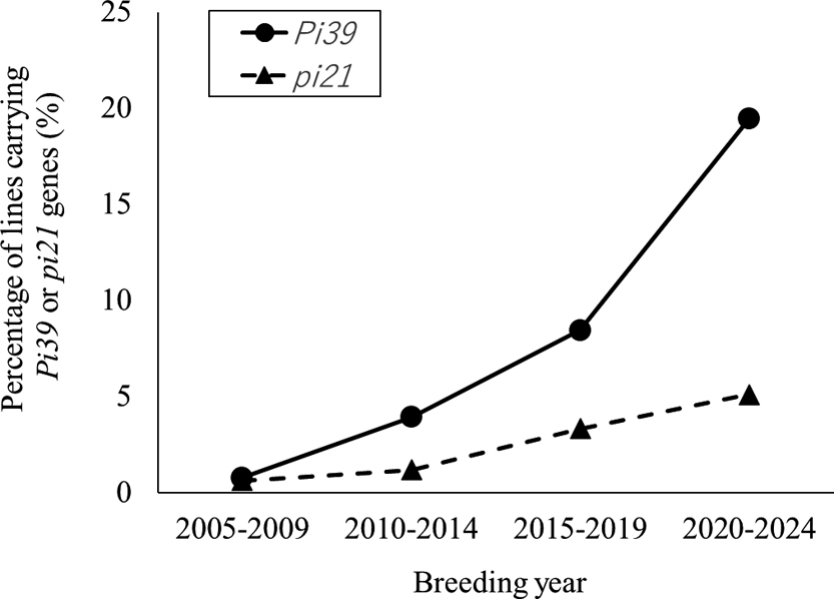
Temporal trends in the percentage of newly developed rice breeding lines carrying *Pi39* or *Pi21* genes by NARO and prefectural research stations nationwide. The data were compiled by NARO based on breeding line information collected from NARO and prefectural research stations across Japan. The total number of newly developed rice breeding lines was 666 during 2000–2004, 639 during 2005–2009, 585 during 2010–2014, 449 during 2015–2019, and 293 during 2020–2024.

Two leaf blast field resistance genes with high efficacy and non-race-specific action have been identified: *pi21* (Fukuoka *et al.* 2009), derived from the upland rice cultivar ‘Sensho’, and *Pi3*9 (Terashima *et al.* 2008), originating from a Chinese landrace cultivar. Although poor eating quality is closely linked to *pi21* in the donor cultivar, MABC has enabled the introgression of *pi21* while retaining the favorable allelic sequence of high-eating-quality cultivars within a region less than 2.4 kb downstream (Fukuoka *et al.* 2009). Following this approach, the blast-resistant cultivar ‘Tomohonami’ (Saka *et al.* 2010), derived from the high-quality cultivar ‘Koshihikari’ through the introgression of *pi21*, was successfully developed. Since then, MAS has been used to introduce *pi21* into other elite cultivars (Matsumoto *et al.* 2023, Murata *et al.* 2022), further expanding the potential for combining disease resistance with desirable eating quality. *Pi39* was mapped through genetic analysis using the Aichi Prefecture-bred cultivar ‘Mineharuka’ (Saka *et al.* 2007). Since its localization, MAS has been actively used to introduce *Pi39*, leading to the development of blast-resistant cultivars such as ‘Eminoaki’ (Kaji *et al.* 2017). Fig. 1 shows temporal trends in the percentage of newly developed rice breeding lines carrying *Pi39* or *pi21* genes by National Agriculture and Food Research Organization (NARO) and prefectural research stations nationwide. The proportion of lines carrying these genes has increased over time, reaching 19.5% for *Pi39* and 5.1% for *pi21* during 2020–2024, indicating active use of both genes through MAS for blast resistance improvement.

It has been suggested that pyramiding field resistance genes with different resistance mechanisms into a single rice cultivar could help improve the stability and durability of resistance to rice blast disease (Fukuoka *et al.* 2015). Based on this concept, cultivars such as ‘Tachiharuka’ (Sakai *et al.* 2014) and ‘Mineasahi SBL’ (Suzuki *et al.* 2017), both carrying *Pb1* and *Pi39*, as well as the strategically developed line ‘Chubu-mochi 136’ carrying *Pb1*, *Pi39*, and *pi21*, have been bred using DNA markers. In addition to blast resistance, all of these cultivars carry the rice stripe disease resistance gene *Stvb-i*. Among these cultivars, ‘Mineasahi SBL’ has been widely adopted in mountainous areas of Aichi Prefecture, where pesticide-free cultivation practices have been implemented for both rice blast and rice stripe disease.

### Improvement of resistance to rice stripe disease

Rice stripe disease is caused by rice stripe virus, which is transmitted by the small brown planthopper (*Laodelphax striatellus*). As climate change is expected to shift the regions where the small brown planthopper can thrive (Yamamura and Yokozawa 2002), the development of resistant cultivars is increasingly recognized as a key strategy for reducing rice stripe disease risks. Conventionally, resistance to rice stripe disease has been evaluated through a bioassay involving the rearing of the small brown planthopper and inoculation of rice plants, a process that demands considerable labor and resources and over a month to complete. Consequently, the introduction of resistance to rice stripe disease attracted early attention to the use of MAS. By the early 21st century, efforts had already begun to design markers and conduct selection targeting the *Stvb* locus, which contains an effective resistance gene derived from ‘Modan’ (Hayano-Saito *et al.* 2000). In the early stages of marker development, only flanking markers were available, which did not always allow for accurate identification of resistance. However, with advances in genetic analysis, a more precise marker, ST71, was developed based on sequence variation in the fourth intron of the *Stvb* gene, enabling reliable detection of rice stripe disease resistance (Hayashi *et al.* 2022). The ST71 marker enables the evaluation of resistance by distinguishing among multiple alleles at the *Stvb* locus: *Stvb-i* (from the *indica* cultivar ‘Modan’), *Stvb* (from *japonica* upland rice), and *Stvb-o* (from *Oryza officinalis*), in comparison with the susceptibility allele *stvb-j* (from *japonica* paddy rice). These markers have replaced the conventional bioassay, enabling the determination of resistance status within several hours. Furthermore, the ST5-BO marker was designed to clearly distinguish resistance alleles derived from foreign cultivars or wild species, such as *Stvb-i* and *Stvb-o*, from the *Stvb* allele originating from Japanese upland rice. Both ST71 and ST5-BO are highly reliable, and the acquisition of polymorphism data for these markers has been officially recognized as valid supporting evidence for the presence of rice stripe disease resistance genes in the context of varietal registration (MAFF 2024). In addition to *Stvb*, Japanese upland rice cultivars also carry the *Stva* gene, for which molecular markers have been developed through genetic mapping (Maeda *et al.* 2004, 2006). While *Stvb-i* carrying donors are commonly used in breeding for rice stripe disease resistance, the cultivar ‘Koshihikari Kinchushi SBL1’ was developed by introducing both *Stva* and *Stvb* from upland rice using MAS. The *Stva* gene is thought to enhance the effect of *Stvb* by moderating symptom severity rather than reducing the number of infected plants (Maeda *et al.* 2006). Owing to its relatively weak effect and distinct mode of action, *Stva* has rarely been used alone in breeding programs.

**Fig. 2.**
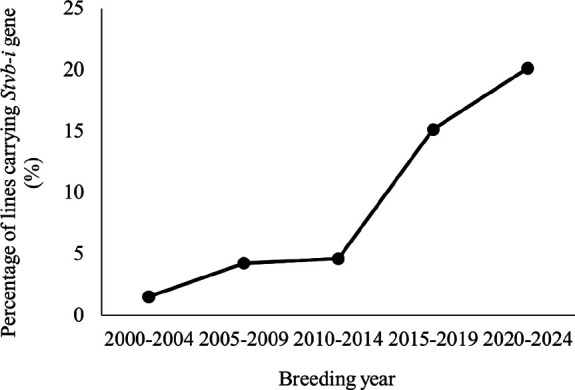
Temporal trends in the percentage of newly developed rice breeding lines carrying the *Stvb-i* gene by NARO and prefectural research stations nationwide. The data were compiled by NARO based on breeding line information collected from NARO and prefectural research stations across Japan. The total number of newly developed rice breeding lines was 639 during 2005–2009, 585 during 2010–2014, 449 during 2015–2019, and 293 during 2020–2024.

*Stvb-i* and *Pb1*, both inherited from the *indica* rice cultivar ‘Modan’, are genetically linked with a recombination frequency of 5.2% (Fujii *et al.* 1999). This close linkage implies that the genes have often been co-selected during breeding. Recent cultivars that have expanded in production area, such as ‘Niji-no-kirameki’ (Nagaoka *et al.* 2020), frequently carry both genes, reflecting a breeding trend toward enhanced disease resistance. No breakdown of resistance conferred by *Stvb-i* has been reported. Its use in MAS has become widespread in Japanese rice breeding due to its efficiency in selection and contribution to long-term disease control. Fig. 2 shows temporal trends in the percentage of newly developed rice breeding lines carrying *Stvb-i* by NARO and prefectural research stations nationwide. The proportion has steadily increased, reaching 20.1% during 2020–2024, indicating active use of the gene for rice stripe virus resistance. The use of resistant cultivars developed through MAS has contributed to reduced insecticide application, thereby lowering cultivation costs and supporting environmental conservation.

### Improvement of resistance to other rice diseases

Brown spot (BS), caused by *Bipolaris oryzae*, and bacterial seedling rot (BSR) and bacterial grain rot (BGR), both caused by *Burkholderia glumae*, are increasingly favored by rising temperatures (Ham *et al.* 2011, Savary *et al.* 2011). With recent advancements in the assessment of disease susceptibility, the identification of multiple QTLs associated with resistance to these pathogens has been progressing (Mizobuchi *et al.* 2016).

In the case of BS, a QTL analysis using progenies derived from the highly resistant cultivar ‘Tadukan’, originally from the Philippines, and the Japanese cultivar ‘Hinohikari’ led to the identification of *bsr1*, a gene that significantly contributes to enhanced disease resistance (Matsumoto *et al.* 2021, Sato *et al.* 2008). *bsr1* was introduced into the cultivar ‘Mienoyume’, resulting in ‘Mienoyume BSL’. This cultivar exhibited stable resistance to BS and superior agronomic performance, including a 6% higher yield and larger grain width under mild BS conditions, and a 29% higher yield with significantly lower disease scores under severe BS pressure (Matsumoto *et al.* 2021). It was also resistant to multiple BS isolates. ‘Mienoyume BSL’ has since replaced ‘Mienoyume’ and is now widely cultivated in Mie Prefecture.

For BSR, a QTL analysis using chromosome segment substitution lines (CSSLs) derived from the resistant cultivar ‘Nona Bokra’ and the susceptible cultivar ‘Koshihikari’ led to the identification of *RBG1*, located on chromosome 10, which suppresses BSR symptoms (Mizobuchi *et al.* 2013). Mizobuchi *et al.* (2023) reported that *RBG1* encodes a mitogen-activated protein kinase kinase kinase (MAPKKK) that is involved in plant defense signaling and phosphorylates OsMKK3. They also demonstrated that the function of *RBG1* was clearly mediated by the negative regulation of abscisic acid. A near-isogenic line (NIL), *RBG1*-NIL, was produced by introducing the genomic region containing *RBG1* into ‘Koshihikari’; its evaluation confirmed that *RBG1* effectively decreased the severity of BSR (Mizobuchi *et al.* 2020) and was also effective against bacterial seedling blight caused by *Burkholderia plantarii* (Mizobuchi *et al.* 2023). But even though the same organism (*Burkholderia glumae*) causes both BSR and BGR in rice, the latter is not controlled by *RBG1*. A major QTL for BGR resistance, *RBG2*, was mapped on the long arm of chromosome 1 in backcross inbred lines derived from a cross between ‘Kele’, a resistant traditional lowland cultivar (*indica*) that originated in India, and ‘Hitomebore’, a susceptible modern lowland cultivar (temperate *japonica*) from Japan (Mizobuchi *et al.* 2015). The correlation between resistance scores for BSR and BGR was low, and to date, no QTLs have been reported that confer resistance to both diseases simultaneously (Mizobuchi *et al.* 2020). Because disease symptoms of BSR and BGR appear in different tissues and at different stages, it may be difficult to control both with a single gene. However, since both diseases are caused by *Burkholderia glumae*, we hope to discover a gene that can control the movement of this bacterium in the future.

Sheath blight caused by *Rhizoctonia solani* is also considered to be promoted under high-temperature conditions (Mizobuchi *et al.* 2016). The severity of sheath blight is strongly influenced by heading date and plant height above the water line, which poses a challenge in identifying QTLs that confer physiological resistance to the disease (Pinson *et al.* 2005, Zeng *et al.* 2015). Although no major resistance genes have been identified for this disease, several cultivars have been reported to exhibit field resistance (Li *et al.* 1995, Sato *et al.* 2004, Zeng *et al.* 2015). At present, MAS for improving sheath blight resistance has made little progress. However, as research on genetic resources exhibiting field-level resistance progresses, the development of markers with high trait-introduction efficiency is expected to advance.

Another disease of rice, bacterial leaf blight, caused by *Xanthomonas oryzae pv. oryzae*, tends to cause more severe damage in other countries than in Japan. Numerous resistance QTLs have been identified globally, and efforts have been made to enhance disease resistance through pyramiding of these QTLs (Pradhan *et al.* 2023, Shanti *et al.* 2001, Singh *et al.* 2001). If climate change leads to a significant increase in damage caused by the disease in the future, it is expected that resistance enhancement with foreign genetic resources will be promoted even within Japan.

### Improvement of resistance to sap-sucking insect pests

The brown planthopper (*Nilaparvata lugens*; BPH) is a major insect pest of rice in Japan. It migrates from Southeast Asia to Japan on the westerly winds and feeds on rice plants by sucking sap, often causing symptoms such as “hopperburn”. In years when BPH outbreaks are severe, economic losses exceeding 10 billion yen have been reported, particularly in Kyushu region (Sanada 2020). The primary method for controlling BPH is the application of chemical pesticides. However, excessive and indiscriminate use of chemicals leads to environmental pollution, kills natural enemies of the target pests, and the emergence of pesticide-resistant BPH populations, ultimately resulting in a resurgence of BPH outbreaks (Lakshmi *et al.* 2010). Therefore, enhancing plant resistance to BPH is increasingly considered a promising alternative strategy. Since the 1960s, scientists have been systematically screening and identifying many BPH-resistant materials: to date, 70 BPH-resistance genes/QTLs (50 genes and 20 QTLs) have been identified in rice (Yan *et al.* 2023). Some wild rice species, such as *Oryza officinalis*, exhibit strong resistance to multiple biotypes of BPH, and several genes effective in enhancing this resistance have been identified (Hu *et al.* 2016). Using MABC, Japanese researchers developed a BPH-resistant rice cultivar named ‘Kanto BPH1’ in 2007 by introducing the resistance gene *bph11*, derived from *Oryza officinalis*, into ‘Hinohikari’, the leading cultivar in Kyushu region (Ando 2014). ‘Kanto BPH1’ attracted attention for its potential to reduce pesticide use. However, BPH biotypes capable of overcoming *bph11* resistance were later detected. In response, efforts were made to develop new lines incorporating a different QTL, *Qbp4*, also derived from *Oryza officinalis*, into ‘Kanto BPH1’ (Ando 2014). Nevertheless, a rice variety with definitive and durable BPH resistance has yet to be achieved in Japan. Meanwhile, several studies have reported cases in which BPH resistance was enhanced by pyramiding multiple resistance genes (Hu *et al.* 2013, Qiu *et al.* 2012, Tamura *et al.* 2014). The elite IRRI variety ‘IR64’ carries the *Bph1* gene; however, it has been reported to exhibit more durable and stable resistance to BPH than ‘IR26’, another *Bph1*-carrying variety. This enhanced resistance is attributed to the presence of additional minor QTLs beyond *Bph1* (Alam and Cohen 1998), illustrating the importance of paying attention to minor genes that may enhance resistance when pyramiding resistance loci. Further enhancement of BPH resistance is expected through detailed functional analysis of resistance genes and the effective use of MAS utilizing genetic resources from outside Japan.

Like BPH, the white-backed planthopper (*Sogatella furcifera*; WBPH) also feeds on rice phloem sap and causes damage to the crop. Many *japonica* rice cultivars in Japan exhibit ovicidal resistance to WBPH, which has helped prevent serious damage (Sogawa 1991, Yamasaki *et al.* 2003). On the other hand, some widely grown *japonica* cultivars, such as ‘Asahinoyume’, exhibit relatively weak resistance to planthoppers. In addition, as new cultivars with *indica* lineage—mainly developed for forage rice—have become more widely grown, there is growing concern that WBPH infestation may lead to yield loss. A gene with ovicidal activity against WBPH, designated *Ovc*, was identified using NILs with reciprocal genetic backgrounds of the non-ovicidal *indica* variety ‘IR24’ and the ovicidal *japonica* cultivar ‘Asominori’ (Yamasaki *et al.* 2003). DNA marker analysis of the *Ovc* locus in ‘Asahinoyume’ revealed that it carries the same haplotype as ‘IR24’, which lacks the *Ovc* gene, thereby clarifying the cause of its weak resistance (Nakamura *et al.* 2009). Furthermore, gene markers associated with the *Ovc* locus have enabled the introduction of ovicidal resistance into high-yielding *indica* cultivars, making it a viable strategy for effective pest control. As climate change adaptation and improved production efficiency become increasingly important, the cultivation of varieties with *indica* lineage is expected to expand. Consequently, MAS targeting *Ovc* may play an increasingly important role in future breeding programs.

The green rice leafhopper (*Nephotettix cincticeps* Uhler; GRH) is a significant insect pest of cultivated rice in the temperate regions of Asia (Ghauri 1971). GRH damages susceptible rice cultivars by feeding on their sap and transmits viruses that cause rice dwarf disease. To date, six genes and two QTLs conferring resistance to GRH have been identified in cultivated rice and its wild relatives (Kham *et al.* 2024). In Japan, NILs of the rice cultivar ‘Kinuhikari’, developed by introducing the resistance genes *Grh1*, *Grh2*, *Grh3*, or a combination of *Grh2* and *Grh4*, have been reported to be resistant to GRH (Hirae 2010). However, the resistance of these lines may vary depending on the local biotypes of GRH. In addition, attention must be paid to the potential breakdown of resistance over time.

While the domestic occurrence of the GRH has been declining, stink bugs that damage rice grains have increased in abundance in Japan since the 2000s (Sugiura and Nakamura 2023, Yamashita 2008). These pests have become a significant issue owing to their impact on rice quality and yield, causing discolored grains and sterile spikelets that reduce the appearance and productivity of brown rice. Possible causes include the proliferation of Poaceae weeds in abandoned farmlands (Ito 2004), as well as effects of climate warming such as an increase in the number of stink bug generations and a decrease in winter mortality rates (Kiritani 2007). Mitigation methods such as Poaceae weed management and chemical control are commonly employed, but costly. Consequently, the development of rice cultivars resistant to rice stink bugs is highly desirable, but breeding research on stink bug resistance remains limited on a global scale. Nevertheless, recent studies have made progress in identifying resistant germplasm and elucidating the mechanisms of resistance, paving the way for the development of resistant cultivars (Sugiura and Nakamura 2023). Cultivars such as ‘Milyang 44’ and ‘CRR-99-95W’ have been identified as resistant to rice stink bug species with different feeding behaviors, including *Leptocorisa chinensis*, which targets the hook region of rice spikelets, and *Nezara viridula*, which exhibits indiscriminate feeding (Sugiura *et al.* 2017). Further investigation suggests that the resistance in these cultivars is associated with structural traits such as cell wall thickness in the sclerenchyma cells of the spikelets (Nakamura *et al.* 2020, Sugiura *et al.* 2022). Based on this finding, future QTL analyses related to resistance against rice stink bugs are anticipated, followed by the development of DNA markers for the effective introduction of these traits into rice cultivars.

## Application of DNA markers for the improvement of quality in rice as a food resource

### Improvement of eating quality

The most effective and practical method for evaluating overall eating quality in rice breeding is the sensory test. However, this method is limited to advanced generations of breeding materials, as it requires large quantities of grain as well as significant time and labor (Ando *et al.* 2010). Therefore, there is considerable interest in introducing MAS for breeding rice with high eating quality. Japanese consumers tend to prefer rice with a moderately sticky and soft texture, as exemplified by the popular cultivar ‘Koshihikari’. Amylose content (AC) is negatively correlated with rice stickiness and has a significant impact on eating quality (Li *et al.* 2023). The *Waxy* (*Wx*) gene, encoding granule-bound starch synthase I, is the primary determinant of AC in rice (Su *et al.* 2011). Multiple alleles at the *Wx* locus influence AC: *wx* in glutinous rice leads to extremely low AC levels (near 0%), *Wxa* in *indica* cultivars results in relatively high AC levels (typically >20%), and *Wxb* in *japonica* cultivars including ‘Koshihikari’ produces moderate AC levels (approximately 14–18%) (Sano *et al.* 1986, Yamanaka *et al.* 2004). Additionally, novel *Wx* alleles have been identified that reduce AC compared to standard cultivars. *Wx-mq* was identified in a low-amylose cultivar, ‘Milky Queen’, which was developed through mutagenesis of ‘Koshihikari’ (Sato *et al.* 2001, 2002). Introduction of *Wx-mq* reduces AC to approximately 10%, leading to a characteristic opaque appearance of the rice grains. Low-amylose rice exhibits high stickiness compared to conventional high-eating-quality rice cultivars. Moreover, low-amylose rice tends to maintain its softness even after cooling, which suggests its potential suitability for processed rice products that require extended shelf life, such as chilled packaged rice. Owing to its distinctive characteristics, ‘Milky Queen’ has become a well-known rice cultivar. Subsequently, MAS targeting the *Wx-mq* allele has been used to develop new cultivars with similar traits, such as ‘Himegonomi’ (Iida *et al.* 2011). Another gene capable of achieving an AC comparable to that of ‘Milky Queen’ is *du*, which is found in ‘Aya’ (Kunihiro *et al.* 1993), the first low-amylose rice cultivar developed in Japan. However, *du* confers greater sensitivity to temperature during the grain-filling stage, resulting in less stable AC than in ‘Milky Queen’ (Tateyama *et al.* 2005). Consequently, when breeding rice cultivars with similarly low AC, the introduction of *Wx-mq* is generally preferred.

On the other hand, low-amylose rice with an opacity level comparable to that of glutinous rice tends to exhibit a distinctive aroma, which may be undesirable to certain consumers (Tanno *et al.* 1997). For household use as table rice, there have been suggestions that cultivars with an AC exceeding 13%, which prevents grain opacity in brown rice, are more suitable than those with around 10% AC. In pursuit of genes capable of resulting in slightly higher AC than that conferred by *Wx-mq*, research efforts have continued, leading to the identification of *Wx1-1* (Ando *et al.* 2010). *Wx1-1* is a gene derived from ‘Hokkai 287’, a somaclonal variant line of the cultivar ‘Kirara 397’. The first registered cultivar carrying this gene is ‘Oborozuki’ (Ando *et al.* 2007), which is a progeny of ‘Hokkai 287’. In Hokkaido region, where the cultivar was developed, the average AC of ‘Oborozuki’ is approximately 14%, and its brown rice has minimal opacity. Furthermore, fluctuations in AC in response to grain-filling temperature are smaller in ‘Oborozuki’ than in ‘Aya’. The eating quality of ‘Oborozuki’ significantly improved the perception of rice grown in Hokkaido region, which had previously been considered inferior in taste owing to high amylose levels associated with low maturation temperatures. ‘Yumepirika’ (Ozaki *et al.* 2018) was subsequently developed in Hokkaido region from the progeny of ‘Hokkai 287’, inheriting the same *Wx1-1* allele and the low-amylose trait similar to that of ‘Oborozuki’. ‘Yumepirika’ is regarded as a moderately high-yielding cultivar and is currently cultivated on approximately 24,000 hectares. Thus, *Wx1-1* has gained a strong reputation in Hokkaido region as a gene that improves eating quality. Elite cultivars such as ‘Sansanmaru’ have been successfully developed using the *Wx1-1* allele, assisted by DNA markers (Matsuba *et al.* 2025).

Furthermore, a QTL designated *qAC9.3*, which originates from ‘Hokkai PL9’ and enables more precise regulation of AC than *Wx1-1*, has been identified on chromosome 9 (Ando *et al.* 2010). This locus is genetically distinct from the *Wx* locus located on chromosome 6. While *Wx1-1* reduces AC by approximately 7.8%, *qAC9.3* has a more subtle effect, lowering AC by only about 2.6%. The *Wx1-1* allele and the ‘Hokkai PL9’ allele of *qAC9.3* showed an additive effect in decreasing AC. In Saitama Prefecture, where ripening temperatures are relatively high, more delicate regulation of AC is required to improve the eating quality of extremely late-maturing cultivars than that is needed in cooler regions such as Hokkaido. In response to this need, ‘Kuiku 162’, which possesses the *qAC9.3* allele, has been used as a donor for the development of new lines with improved eating quality (Munakata *et al.* 2021). Although the lines developed through this approach were not officially registered as cultivars because they had reduced yield, they were evaluated as valuable genetic resources in terms of eating quality. Several other QTLs involved in the fine regulation of AC have been reported (Li *et al.* 2003, 2011, Septiningsih *et al.* 2003, Takemoto-Kuno *et al.* 2015, Takeuchi *et al.* 2007, Wan *et al.* 2003, 2004). The MAS of such QTLs is expected to further facilitate the development of high-eating-quality lines adapted to diverse climatic conditions.

In addition to the texture controlled by AC, increasing attention has been paid to the taste component of eating quality. QTLs associated with sensory evaluation of good eating quality have been reported for ‘Koshihikari’ (Takeuchi *et al.* 2007, 2008, Wada *et al.* 2008) and ‘Sakihikari’ (Kobayashi and Tomita 2008). Notably, a QTL named *qOE3*, located on the short arm of chromosome 3 has been consistently detected across studies. This QTL exerts a major influence on sensory evaluation scores, particularly taste, but is independent of AC. Among high-yielding cultivars developed from *indica* rice, some possess AC levels comparable to those of Japanese high-quality cultivars yet still fail to satisfy the taste preferences of Japanese consumers. Such QTLs may improve the eating quality of these cultivars independently of AC through MAS.

Although strongly aromatic rice is not widely preferred in Japan, fragrance is a key determinant of market value internationally. Variety groups such as Basmati and Jasmine rice occupy premium positions in global markets owing to their distinctive aroma (Pachauri *et al.* 2010). The compound 2-acetyl-1-pyrroline (2-AP) is considered the major contributor to the distinctive fragrance of both Basmati and Jasmine rice (Buttery *et al.* 1982). The *fgr* gene located on chromosome 8, which regulates the biosynthesis of 2-AP, encodes the enzyme betaine aldehyde dehydrogenase 2 and plays a decisive role in determining whether a rice cultivar is aromatic (Shi *et al.* 2008). In future breeding programs aimed at developing export-oriented cultivars with added value for international markets, molecular markers targeting this gene could be used to introduce fragrance into high-eating-quality cultivars.

### Improvement of grain quality for rice-based food products

Glutinous rice, which contains almost no amylose, is commonly used in the production of mochi (rice cakes) and traditional Japanese sweets such as ‘Daifuku’ and ‘Ohagi’. For production of sweets, glutinous rice cultivars with low hardening tendencies are preferred (Suzuki *et al.* 2019). To mitigate hardening, additives such as amylase and sugars are sometimes incorporated during processing. The molecular structure of amylopectin significantly influences the hardening properties of glutinous rice. When the degree of polymerization of glucose units in amylopectin decreases, the pasting temperature of starch also decreases, resulting in reduced hardening (Igarashi *et al.* 2008).

The upland rice cultivars ‘Hiderishirazu-D’ and ‘Kurmai’ both exhibit low pasting temperatures (Okamoto *et al.* 2013). These cultivars lack starch branching enzyme I (Sbe1) activity in the endosperm during the grain-filling stage and have a high proportion of short chains in amylopectin. Among the progeny lines derived from a cross between ‘Kurmai’ and a Japanese glutinous rice cultivar ‘Mangetsumochi’, those lacking Sbe1 activity exhibit reduced glutinous rice hardening. Furthermore, this trait has been shown to be associated with a recessive mutation at the *Sbe1* locus located on chromosome 6. Building on these findings, a molecular marker was developed based on specific changes in the promoter region of the mutant *sbe1* gene. By using this marker, a new glutinous cultivar, ‘Yawakoimochi’, carrying the *sbe1* gene derived from ‘Hiderishirazu-D’, was successfully bred (Suzuki *et al.* 2019). Japanese sweets made without additives but using this cultivar achieved the intended increase in softness, and more than 88% of general consumers rated their taste as superior to that of prototypes made with ‘Hiyokumochi’, which possesses a functional *Sbe1* allele (Suzuki *et al.* 2019).

Sbe1 deficiency is also beneficial in other ways, as it lowers the pasting temperature of rice flour during cooking and reduces the hardening of rice flour bread (Aoki *et al.* 2015). ‘Yawaramaru’ is a new rice cultivar developed for bread-making by introducing *sbe1* via MAS. Moreover, Sbe1 deficiency might also mitigate the adverse effects of climate warming on the processing qualities of rice. For example, high air temperature during grain filling reduces the enzyme digestibility of steamed rice in Sake brewing (Okuda 2019) and accelerates the hardening of processed foods such as rice flour bread (Aoki *et al.* 2018), both because of the decrease in the short-chain ratio of amylopectin at high air temperatures (Umemoto *et al.* 2022). Thus, genetic resources that increase the short-chain ratio of amylopectin without decreasing the grain weight, such as the Sbe1-deficient cultivars, could be useful in future rice breeding.

To develop rice intended for flour-based products, a large quantity of rice is typically required for testing, making it difficult to evaluate processing suitability until later generations when sufficient material has been multiplied. However, the integration of MAS has significantly improved the efficiency of selection. High-AC rice cultivars carrying *Wxa*, which tend to harden and are therefore unpopular for table use in Japan, are well-suited for rice noodle processing. ‘Koshinokaori’ was developed using MAS, by which the *Wxa* gene from the Indian landrace ‘Surjamukhi’ was introduced into ‘Kinuhikari’. ‘Koshinokaori’ contains more than 30% amylose and was registered as Japanese first rice cultivar suitable for rice noodle production (Sasahara *et al.* 2013). Noodles made from ‘Koshinokaori’ differ from those made with common rice cultivars in that they do not stick together and offer a smooth, pleasant texture when swallowed. MAS was also effectively used to introduce the *Wxa* allele in the development of ‘Ajianokaori’ (Matsushita *et al.* 2020), another cultivar for noodle production that was bred to surpass ‘Koshinokaori’ in yield while retaining its desirable AC levels. In contrast, for rice bread production, no specific gene has yet been identified that determines the optimal AC of 20–23%, which is considered ideal for this purpose. Further research is needed, and the development of a comprehensive marker set for future selection is highly anticipated.

## Application of DNA markers for enhancing tolerance to abiotic stresses

### Enhancement of high-temperature tolerance

In brown rice exposed to high temperatures during the grain-filling stage, insufficient starch supply relative to morphological development leads to an increased occurrence of chalky grains, which exhibit partial or complete opacity (Morita 2008). Chalky grains are undesirable in rice production, as they break easily during milling and result in lower milling efficiency. Some *indica* cultivars possess genetic regions that reduce the occurrence of chalky grains under high temperatures, and genetic analyses using both *indica* and *japonica* cultivars have identified multiple QTLs that contribute to enhanced high-temperature tolerance. Among the identified loci, *Apq1* (also known as *SUS3*), located on chromosome 7 and encoding the enzyme sucrose synthase 3, has attracted considerable attention owing to its particularly strong effect, as revealed through genetic analysis of CSSLs derived from the *indica* cultivar ‘Habataki’ and the *japonica* cultivar ‘Koshihikari’ (Murata *et al.* 2014, Takehara *et al.* 2018). Following the discovery of this gene, a new cultivar named ‘Fu-Fu-Fu’ was developed in Toyama Prefecture through MABC, in which *Apq1* was pyramided with *sd1*, *Pita-2*, and *pi21* in the genetic background of ‘Koshihikari’ (Murata *et al.* 2022). This cultivar improves upon the weaknesses of ‘Koshihikari’ by enhancing high-temperature tolerance, reducing plant height, and increasing resistance to rice blast disease. This cultivar has been improved from the “slightly weak” level of high-temperature tolerance seen in ‘Koshihikari’ to the “strong” level, the highest among domestic varieties, while maintaining comparable yield and eating quality to ‘Koshihikari’. In Toyama Prefecture, where the summer of 2023 was exceptionally hot, the proportion of first-grade ‘Koshihikari’ dropped significantly to 48%, while ‘Fu-Fu-Fu’ maintained a high rate of 93%; based on this performance, it is planned to expand the cultivation area of this variety 10,000 hectares by 2028 (Toyama Prefecture 2024). Kobayashi *et al.* (2016) reported that the seed dormancy-related gene *Sdr4* (Sugimoto *et al.* 2010), which confers pre-harvest sprouting resistance and originates from the Indian cultivar ‘Kasalath’, also contributes to high-temperature tolerance. ‘Kanto IL32’, a NIL produced by introducing *Sdr4* into ‘Koshihikari’, may serve as a promising breeding material for simultaneous improvement of pre-harvest sprouting resistance and high-temperature tolerance. Three QTLs that increase the percentage of perfect grains without significantly affecting heading date or yield (relative to ‘Koshihikari’) have been identified from CSSLs developed by introducing chromosome segments from the *indica* cultivars ‘IR64’ and ‘Nava’ into the ‘Koshihikari’ genetic background (Fukuda *et al.* 2025). These genetic resources are expected to become powerful tools for introducing high-temperature tolerance through detailed mapping and pyramiding by MAS in the future. In addition, several QTLs associated with high-temperature tolerance have been identified from the *japonica* cultivars ‘Hanaechizen’ and ‘Eminokizuna’, which are relatively tolerant to high temperature (Kobayashi *et al.* 2013, Nagaoka *et al.* 2017). These QTLs are also expected to contribute to countermeasures against rapidly progressing global warming through marker-assisted pyramiding.

High temperatures during the grain-filling period promote not only the occurrence of chalky grains but also the development of cracked grains (Nagata *et al.* 2004, Takahashi *et al.* 2002). Timely harvesting is important for preventing grain cracking; however, it is expected that more fields will face difficulties in harvesting at the optimal time owing to the concentration of heading dates caused by the progression of global warming and the expansion of cultivated area per farming entity (Matsumura and Yamaguchi 2006), so the development of cultivars that can suppress the occurrence of cracked grains is increasingly needed. Variation in the incidence of grain cracking has been observed among rice cultivars, and in regions such as Tohoku, Hokuriku, and western Japan, high-temperature tolerance is assessed using region-specific standard cultivars (Nakagomi *et al.* 2019, 2020a). Through genetic analysis of progeny derived from a cross between the susceptible cultivar ‘Yamahikari’ and the resistant cultivar ‘Nipponbare’, *qCR2*, a locus involved in grain cracking, was identified on chromosome 2 (Hayashi *et al.* 2017). In addition, the QTL *qCR8-2*, also associated with grain cracking resistance, has been identified on chromosome 8 from CSSLs developed by introducing chromosome segments from the wild rice *Oryza rufipogon* into the genetic background of ‘Itadaki’ (Nakagomi *et al.* 2020b). Such QTL information contributes to the understanding of the physiological mechanisms underlying grain cracking and is expected to be useful for the development of new cultivars with enhanced resistance to grain cracking through the application of MAS.

When rice plants are exposed to extremely high temperatures during flowering, male sterility may occur owing to poor pollen development, failure of anther dehiscence, unsuccessful pollination, and impaired pollen germination (Matsui 2009). Chamber experiments have shown that temperatures exceeding 35°C for a few hours during flowering can induce sterility, with rates increasing as temperatures rise (Satake and Yoshida 1978). In tropical regions, yield losses caused by high-temperature-induced sterility have already been reported (Ishimaru *et al.* 2016b, Matsushima *et al.* 1982, Osada *et al.* 1973). In Japan, although no substantial damage has been reported, abnormally high temperatures exceeding 38°C were recorded in 2007 and 2018, particularly in the Kanto and Tokai regions. Rice flowering during these heat events exhibited higher sterility rates than usual (Hasegawa *et al.* 2011, Yoshimoto *et al.* 2021). Considering future climate trends, Japan is increasingly at risk of high-temperature-induced sterility. The early-morning flowering (EMF) trait is considered effective for heat escape, as it shifts flowering to cooler hours in the morning (Ishimaru *et al.* 2016a, Satake and Yoshida 1978). While flowering time in *Oryza sativa* cultivars is confined to a narrow range of times during the day, wild species such as *Oryza eichingeri* and *Oryza officinalis* show broader variation, including EMF phenotypes (Sheehy *et al.* 2005, 2007). Ishimaru *et al.* (2010) selected the EMF line ‘EMF20’, derived from *Oryza officinalis* (‘Norin 29’ tetraploid / *Oryza officinalis* // ‘Koshihikari’), and demonstrated its ability to avoid high-temperature-induced sterility through EMF. QTL analysis using an F_2_ population and F_3_ lines from a cross between ‘EMF20’ and ‘Nanjing 11’ identified the QTL *qEMF3* as the genetic basis of this trait (Hirabayashi *et al.* 2015). NILs carrying *qEMF3* in the backgrounds of ‘Nanjing 11’ and ‘IR64’ were then developed via MABC. These NILs demonstrated that *qEMF3* contributes to EMF and reduces sterility under high-temperature stress in controlled environments. In Myanmar, a NIL with *qEMF3* in the ‘IR64’ background also showed reduced yield loss under field conditions during extreme heat (Ishimaru *et al.* 2022). In Japan, NILs with accelerated flowering have been developed for four popular cultivars using *qEMF3*-associated markers (Hirabayashi *et al.* 2023); all NILs showed tolerance to high-temperature-induced sterility in growth chambers and greenhouses. The effect of *qEMF3* is considered stable, and its introduction via MAS is expected to enhance heat avoidance in future rice breeding.

An Indian *aus*-type landrace, ‘N22’, has been recognized as a heat-tolerant cultivar that maintains fertilization ability under high-temperature conditions, regardless of flowering time (Hakata *et al.* 2017, Mackill *et al.* 1982, Satake and Yoshida 1978). QTL analysis using F_2_ populations derived from a cross between ‘N22’ and ‘IR64’ revealed several loci associated with enhanced tolerance to heat-induced sterility (Ye *et al.* 2012). Among these, *qHTSF4.1* was also validated through genetic analysis involving another heat-tolerant cultivar, ‘Giza178’, and is considered a promising source for improving heat tolerance in rice at the flowering stage (Ye *et al.* 2015). MAS targeting *qHTSF4.1* is expected to be an important tool for breeding rice cultivars with enhanced heat tolerance during flowering.

### Enhancement of low-temperature tolerance

Rice is most sensitive to low temperatures at the booting stage, and sterility induced by cold stress at this phase is irreversible, directly resulting in yield loss. In cold regions such as Hokkaido, cold tolerance at the booting stage has consistently been a key trait targeted in rice breeding programs. Genetic analysis using ‘Norin PL8’, which possesses high cold tolerance derived from the Indonesian tropical *japonica* cultivar ‘Silewah’, has led to the identification of two QTLs for cold tolerance, *Ctb1* and *Ctb2*, located on chromosome 4 (Saito *et al.* 2001). Analysis using another highly cold-tolerant line, ‘Hokkai PL9’, resulted in the discovery of three QTLs: *qCTB1.1* and *qCTB1.2* on chromosome 1, and *qCTB8* on chromosome 8 (Kuroki *et al.* 2007, 2011). Using MABC, NILs carrying some of these QTLs have been developed in valuable breeding materials such as ‘Hokkai 287’ and ‘Hoshinoyume’, and have been used in Hokkaido region as breeding materials with enhanced cold tolerance (Fujino *et al.* 2014). However, the introduction of *Ctb1* and *Ctb2* in Hokkaido region tends to delay heading by several days, indicating the need to consider genetic background when utilizing these QTLs. In recent years, summer temperatures exceeding 30°C have become increasingly common even in Hokkaido region. Since QTLs related to cold tolerance and those associated with heat tolerance represent different loci and likely function independently, pyramiding these traits using DNA markers may represents an effective strategy for developing rice cultivars capable of maintaining stable yields under sudden climatic fluctuations in northern regions.

### Reduction of contaminant uptake from soil

Food contamination by toxic metals such as cadmium (Cd) poses a significant threat to human health. In accordance with the Codex General Standard for Contaminants and Toxins in Food and Feed (Codex Alimentarius 2024), the maximum permissible level of Cd in polished rice is set at 0.4 mg/kg, a limit that has also been adopted by the Japanese government to ensure food safety. However, when assessed against this safety threshold, certain regions in Japan have been identified where cultivation of common rice varieties requires Cd uptake mitigation strategies. Ishikawa *et al.* (2012) applied carbon ion-beam irradiation to ‘Koshihikari’ and developed three mutant lines with Cd uptake reduced to nearly undetectable levels. Sequence analysis identified *osnramp5* on chromosome 7 as the causal gene. When grown in Cd-contaminated paddy fields, the mutants showed minimal Cd accumulation in grains and no significant agronomic differences from ‘Koshihikari’. One line was registered as the new cultivar ‘Koshihikari Kan No. 1’. Although continuous flooding can suppress Cd uptake, it may increase accumulation of arsenic (As), another toxic element. In Japan, some regions face risks of exceeding safety thresholds for both Cd and As, complicating mitigation strategies. Under aerobic cultivation with alternate wetting and drying and water-saving conditions, ‘Koshihikari’ showed reduced As uptake but increased Cd accumulation. In contrast, ‘Koshihikari Kan No. 1’ suppressed both Cd and As uptake while maintaining acceptable yield levels, demonstrating its potential as a dual-mitigation cultivar under such conditions (Ishikawa *et al.* 2016). These findings suggest that rice lines incorporating the mutant *osnramp5* gene via MAS can simultaneously reduce Cd and As accumulation risks. Currently, the mutant *osnramp5* allele from ‘Koshihikari Kan No. 1’ has been introduced into more than 20 rice varieties through MABC. In Akita Prefecture, a new rice cultivar with low-Cd-uptake trait, ‘Akitakomachi R’, was developed by introducing *osnramp5* into the conventional cultivar ‘Akitakomachi’. This improved line has been widely adopted across the prefecture, replacing the original cultivar in commercial production. Such initiatives are expected to enhance the safety profile of Japanese rice not only domestically but also in international markets.

In contrast to efforts aimed at reducing Cd accumulation in rice, a QTL associated with enhanced Cd uptake, *qCdp7*, has also been identified on chromosome 7 (Abe *et al.* 2011). This QTL is utilized through MAS to develop cultivars for phytoremediation purposes, i.e., for removing Cd from contaminated paddy fields.

### Enhancement of resistance to β-triketone herbicides

In Japan, rice production has exceeded consumer demand for a long time, prompting the development of high-yielding cultivars for feed and processing purposes. Many of these cultivars have been derived from *indica*-type genetic backgrounds, which sometimes confer sensitivity to β-triketone herbicides. These herbicides inhibit 4-hydroxyphenylpyruvate dioxygenase, and the sensitivity of some cultivars to β-triketone herbicides poses a barrier to adoption of the cultivars in agricultural practice. Maeda *et al.* (2019) identified the *HIS1* gene, conferring resistance to these herbicides, and the use of DNA markers now enables the breeding of resistant cultivars through MAS. This represents a practical example of how MAS can be rapidly applied to overcome barriers associated with the introduction of previously underutilized genetic resources.

## Application of DNA markers for improvement of other agronomically important quantitative traits

Important agronomic traits such as heading date and yield are representative quantitative traits, determined by the combined effects of numerous loci with small individual contributions distributed across the genome. For these traits as well as the ones described above, many QTLs with relatively large effects have been identified through extensive QTL analyses, and marker-assisted improvement has been implemented for some of them.

Takeuchi *et al.* (2006) developed a series of NILs by introducing four QTLs associated with heading date, identified from the progeny of a cross between ‘Koshihikari’ and ‘Kasalath’, into ‘Koshihikari’ through MABC. These NILs were used to experimentally demonstrate that rice heading date can be controlled through marker-assisted breeding, with heading dates ranging from approximately 15 days earlier to about 10 days later than ‘Koshihikari’. Although these NILs closely resembled ‘Koshihikari’, they sometimes exhibited variation in certain morphological traits such as culm length and panicle number, depending on the growth environment. The approach of modifying heading date through MAS has also been applied to the high-value, low-amylose cultivar ‘Milky Queen’. Using DNA markers linked to the loss-of function indica allele of *Hd1* (conferring earlier heading; Yano *et al.* 2000), an early-heading cultivar named ‘Milky Summer’ was developed, while MAS for the *indica* allele of *DTH8* (conferring later heading; Wei *et al.* 2010) led to the creation of a late-heading cultivar ‘Milky Autumn’. Although these cultivars exhibit some morphological differences from the original ‘Milky Queen’, they have successfully inherited its desirable traits, such as low AC. These cultivars are being used for sowing-date diversification in large-scale farming operations and for expanding production into regions where ‘Milky Queen’ was previously difficult to grow owing to heading-date constraints. Genetic markers associated with heading date are particularly useful when crossing cultivars with markedly different maturity periods.

Grain yield is influenced by various factors including sink capacity, source ability, and lodging resistance, and genes and QTLs associated with each of these traits have been extensively investigated. A representative QTL affecting sink capacity, *Gn1a*, was identified on chromosome 1 through genetic analysis using backcross inbred lines derived from a cross between *indica* high-yielding cultivar ‘Habataki’ and *japonica* cultivar ‘Koshihikari’ (Ashikari *et al.* 2005). Its positive effect on grain number was confirmed when the *Gn1a* allele derived from ‘Habataki’ was introduced into ‘Koshihikari’. In the case of another sink capacity gene, *APO1* on chromosome 6, the introduction of the ‘Habataki’-type allele into ‘Koshihikari’ increased the number of grains, particularly on secondary rachis branches, and enhanced culm strength, thereby contributing to yield improvement (Ookawa *et al.* 2010). Through genetic analysis using progeny derived from a cross between ‘Kasalath’ and ‘Koshihikari’, *TGW6* on chromosome 6, which increases grain length and thousand-grain weight, has been identified (Ishimaru *et al.* 2013). Introduction of the *TGW6* allele derived from ‘Kasalath’ into ‘Nipponbare’ increased yield. From a set of CSSLs developed by introducing chromosomal fragments from ‘Takanari’ into ‘Koshihikari’, *MP3*, which affects panicle number, was identified on chromosome 3 (Takai *et al.* 2014); the ‘Takanari’ allele at this locus increases panicle number. Other QTLs harboring naturally occurring variants associated with sink capacity have also been identified outside Japan, including *GNP1* (Wu *et al.* 2016), which affects grain number per panicle; *GW2* (Song *et al.* 2007), which affects grain width and thousand-grain weight; *GS3* (Fan *et al.* 2006, Nan *et al.* 2018), which influences grain length and weight; and *DEP1* (Huang *et al.* 2009), which promotes the formation of erect panicles with high grain density. These QTLs have also been reported to influence grain yield, depending on their allelic variation.

In addition to sink capacity, source ability is also important for grain yield formation. From the perspective of source traits, several QTLs have been identified in high-yielding cultivars such as ‘Takanari’, including *GPS* (Takai *et al.* 2013), which is associated with individual leaf photosynthetic rate; *qCTd11* (Fukuda *et al.* 2018), which enhances photosynthetic activity accompanied by reducing leaf temperature; and *qLIA3* (San *et al.* 2018), which improves light interception by modifying leaf blade inclination angle. Among these, the ‘Takanari’-type allele of *qCTd11* increased grain yield when introduced into ‘Koshihikari’ (Ueda *et al.* 2021).

Because shortening culm length is considered effective for enhancing lodging resistance, the *sd1* gene, located on chromosome 1, has attracted significant attention. It encodes *OsGA20ox2*, which confers a semi-dwarf phenotype and enhances lodging resistance under high fertilizer input. This contributes to increased grain yield and playing a central role in the Green Revolution (Sasaki *et al.* 2002). This gene has already been used in the development of widely adopted cultivars in Japan, such as ‘Koshihikari Tsukuba SD1’ (Wang *et al.* 2005) and ‘Fu-Fu-Fu’ (Murata *et al.* 2022), both developed through MAS. Similar to *sd1*, *OsGA20ox1* on chromosome 3, which is identical to *GNP1*, is another member of the gibberellin 20-oxidase gene family involved in gibberellin biosynthesis. It has also been reported to affect culm length (Oikawa *et al.* 2004) and may contribute to culm shortening and yield improvement. In addition to shortening culm length, strengthening culm structure is also considered effective for improving lodging resistance. Ookawa *et al.* (2010) conducted a genetic analysis using CSSLs developed by introgressing genomic segments from ‘Habataki’, an *indica* cultivar, into the genetic background of ‘Sasanishiki’. They identified two QTLs associated with increased culm strength in the ‘Habataki’ type: *SCM1* on chromosome 1 and *SCM2* on chromosome 6. *SCM1* was found to be effective in increasing culm wall thickness, while *SCM2* was identified as being identical to the *APO1* gene, which exerts pleiotropic effects, including increasing both spikelet number and culm diameter (Ikeda *et al.* 2007). Furthermore, Yano *et al.* (2015) reported two additional QTLs that enhance culm strength: *SCM3* on chromosome 3 and *SCM4* on chromosome 2, both derived from the ‘Chugoku117’, a tropical *japonica* high-yielding line. Among these, *SCM3* was found to be identical to the *MP3* gene, which also regulates tiller number. Ookawa *et al.* (2022) further investigated the effects of pyramiding different combinations of the four QTLs, *SCM1* to *SCM4*, on culm strength. They confirmed that lines carrying a greater number of QTLs exhibited enhanced culm strength, and that the pyramiding of multiple QTLs had an additive effect. In addition to culm strengthening, they also observed an increase in grain number, indicating a pleiotropic effect of these QTLs. Among these pyramided lines, one in which *SCM1*, *SCM3*, and *SCM4* were pyramided into the genetic background of ‘Koshihikari’ showed not only superior lodging resistance but also larger grain size and excellent eating quality, and was subsequently released as the cultivar ‘Sakura Prince’.

The QTLs described above represent only a subset of the genetic resources identified to date. Numerous additional QTLs with the potential contribution to yield improvement have been discovered. However, although numerous genes associated with heading date and grain yield have been identified, MAS for these traits remains relatively limited in domestic breeding programs. This is partly because, unlike traits such as disease resistance or abiotic stress tolerance, which are typically controlled by a few major genes, quantitative traits are influenced by many genes, including those with minor or unknown effects, making it difficult to accurately predict phenotypic outcomes following allele introduction. For example, more than 100 loci, including both major- and minor-effect QTLs, may be involved in the genetic control of heading date (Hori *et al.* 2016). Kitazawa *et al.* (2024) developed a marker set for predicting heading date by using 144 allelic types across 41 QTLs. This marker set enables statistically significant prediction of heading date even for cultivars not included in the training dataset of the prediction model. However, the prediction still shows a deviation of 6.15 to 8.33 days in root mean squared error (RMSE), possibly because of the effects of unknown genes and gene–gene interactions. While this model is considered highly useful for selecting parental lines and narrowing down breeding materials to some extent, it does not provide sufficient predictive accuracy to replace field evaluation of heading date.

In the case of grain yield, numerous minor and yet-to-be-characterized genetic factors are thought to be involved, and the effects of known QTLs can vary depending on the genomic background and production environment. For example, the semi-dwarfing gene *sd1* increased yield when the allele from ‘IR24’ was introduced into ‘Koshihikari’, resulting in the development of ‘Koshihikari Tsukuba SD1’ (Ueda *et al.* 2021, Wang *et al.* 2005). On the other hand, when *sd1* is introduced into short-day *japonica* cultivars with low biomass, lodging resistance is improved, but these cultivars fail to achieve high yield owing to insufficient biomass accumulation (Murai *et al.* 2002).

The introduction of QTLs that enhance sink capacity often increases grain yield under conditions such as elevated CO_2_, which increase source capacity (Nakano *et al.* 2017, Takai *et al.* 2023); however, such effects are not applicable under standard field conditions. Ueda *et al.* (2021) developed NILs by introducing yield-related genes such as *GW2* from high-yielding cultivars into ‘Koshihikari’, but these lines did not show significant yield increases. For example, the *GW2* allele from ‘BG1’ increased panicle weight but caused trade-offs such as reduced panicle number and grain-filling rate. Similarly, the NIL carrying the *GPS* allele from ‘Takanari’ showed improvements in thousand-grain weight and grain-filling rate, but a decrease in spikelets per panicle, resulting in no overall yield gain. These findings suggest that improving either sink capacity or source capacity alone is insufficient to achieve higher yield. Further yield improvement will require coordinated enhancement of both sink and source traits, along with adjustments to canopy architecture, as these components are already well balanced in current major cultivars (Ueda *et al.* 2025). In addition, eating quality and grain appearance must be maintained. Ueda *et al.* (2021) reported that, in an attempt to increase the yield of ‘Akidawara’ (Ando *et al.* 2011), the introduction of the *DEP1* gene into a NIL through MAS resulted in increased rough brown rice yield owing to changes in panicle morphology, but simultaneously caused a significant reduction in the percentage of filled grains. As illustrated by this example, the introduction of QTLs that produce noticeable changes in yield often leads to unintended effects on other traits. To effectively improve quantitative traits through marker-assisted breeding, a deeper understanding of gene–gene interactions, including those involving genes related to other agronomic and quality traits, is essential.

## Accelerated breeding using MAS and generation advancement for efficient trait stacking

Up to this point, we have reviewed the progress and current status of trait improvement through MAS, focusing on individual agronomically important traits. However, in practical rice breeding, it is essential not only to target single traits but also to combine multiple traits without favoring one at the expense of another. Moreover, there is a growing demand to streamline and accelerate the breeding process. This section provides an overview of a practical approach to accelerating breeding through MAS and generation advancement for efficient trait stacking.

In rice breeding using the bulk population method, early-generation populations consisting of hundreds to thousands of individuals must be managed before artificial selection is applied. According to the “Rice Breeding Manual” (Yamamoto *et al.* 1996), the minimum recommended number of individuals in the F_2_, F_3_, and F_4_ generations is 200, 1,500, and 5,000, respectively. But if individuals carrying undesirable alleles can be removed at each generation, the number of plants requiring management can be significantly reduced. At each segregating genetic locus in the F_2_ population, approximately 25% of individuals are homozygous for the desirable allele, 50% are heterozygous, and 25% are homozygous for the undesirable allele. When MAS is applied to a single gene, excluding individuals homozygous for undesirable alleles allows approximately 75% of the population to be advanced. However, when multiple genes are targeted, the proportion of retained individuals decreases sharply. For example, if 10 unlinked genes are selected, only about 5.6% of individuals carry at least one favorable allele at all 10 loci and will be retained (0.75^10^ = 0.056). Similarly, only 0.9% of F_3_ and 0.3% of F_4_ individuals will be selected. Thus, more than 90% of the population from F_2_ to F_4_ may be excluded. Therefore, we recommend a breeding strategy that effectively applies MAS in early generations while maintaining an appropriate number of breeding materials.

**Fig. 3.**
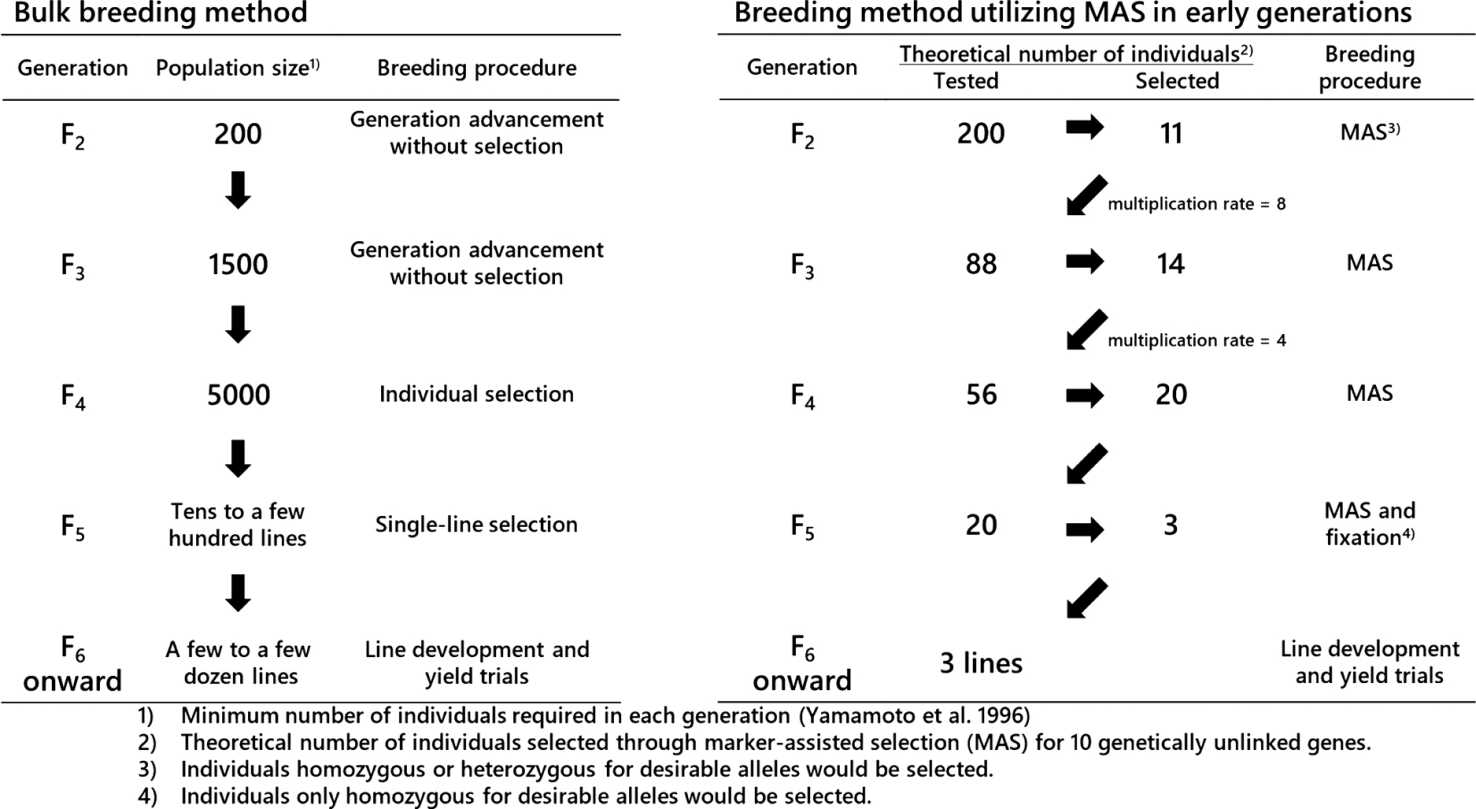
Outline of a breeding method utilizing MAS in early generations and its comparison with the bulk population method. In this hypothetical example, selection is performed by MAS for 10 unlinked genes. Following the description by Yamamoto *et al.* (1996), selection begins from 200 F_2_ individuals. The multiplication rates from F_2_ to F_3_ and F_3_ to F_4_ generations are assumed to exceed those described by Yamamoto *et al.* (1996) in accordance with the actual conditions of generation advancement.

Fig. 3 illustrates our proposed breeding strategy, which utilizes MAS in early generations. In this example, MAS is applied to 10 genes, starting with 200 F_2_ individuals. The multiplication rates from F_2_ to F_3_ and F_3_ to F_4_ generations were set at eight and four, respectively. These values exceed the multiplication rates of 7.5 and 3.3 described by Yamamoto *et al.* (1996). Based on our calculations, fewer than 100 individuals need to be managed in each generation beyond F_3_, and this small population is well-suited for generation advancement under controlled environments. Therefore, MAS in early generations shortens the breeding cycle by several years by eliminating field-level individual selection and single-line selection at the F_4_–F_5_ generations. Selection and multiplication are repeated until the F_4_ generation, and genetic fixation is achieved in the F_5_ generation by excluding heterozygous individuals. In this approach, by incorporating appropriate multiplication steps between generations, we can maintain a number of selected individuals in the F_6_ generation comparable to that of conventional methods. Nevertheless, when selection pressure increases due to the use of a larger number of markers, there is a risk of having an insufficient number of fixed lines for selecting superior traits other than those targeted by the markers.

Following this method, we actually developed a promising line from an F_2_ population derived from an artificial cross. Selection from F_2_ to F_5_ was conducted using 10 markers associated with 7 traits (culm length, resistance to β-triketone herbicides, resistance to blast disease, resistance to rice stripe virus, ovicidal response to whitebacked planthopper, low cadmium accumulation, and grain appearance), which were polymorphic between the parents. The marker-selected lines retained all 10 desirable alleles, and some of them showed no disadvantages in other agronomic traits such as yield and eating quality. Moreover, this attempt resulted in a significant reduction of the breeding period, shortening it from the conventional seven years to approximately five years. To achieve even greater acceleration, integration with rapid-generation advancement systems (Tanaka *et al.* 2016) should be considered. A detailed report on this study is currently in preparation.

## Future perspectives on expanding the use of DNA markers to enhance the efficiency of rice breeding

MAS has become a widely adopted strategy to improve breeding efficiency, particularly for the rapid introduction of target traits in rice. However, MAS is generally applied to a limited set of major genes or QTLs associated with specific traits. Although this approach is suitable for traits controlled by only a few genes, quantitative traits are regulated by both major genes and numerous minor-effect loci; thus, selection strategies that incorporate both types of genetic components are considered more appropriate for improving such traits (Anilkumar *et al.* 2023). In addition, maintaining genetic diversity remains a critical challenge for ensuring continued responsiveness to future demands (Fujino *et al.* 2019).

To address these challenges, it is essential to regularly collect and organize sequence information not only from newly developed cultivars but also from genetic resources such as landraces and wild relatives preserved in gene banks. To facilitate this, searchable databases should be regularly maintained and updated to integrate information on known genes and QTLs, marker polymorphisms present in both newly developed cultivars and diverse genetic resources, and pedigree data. Key resources include RAP-DB (https://rapdb.dna.affrc.go.jp/) (Sakai *et al.* 2013) and Oryzabase (https://shigen.nig.ac.jp/rice/oryzabase/) (Kurata and Yamazaki 2006), which compile gene and QTL data for rice and are regularly updated to support breeding. RAP-DB, in particular, offers a dedicated search page for agronomically important genes. Tools such as TASUKE+ (https://tasuke.dna.affrc.go.jp/) (Kumagai *et al.* 2019), which compares SNP and indel polymorphisms among rice cultivars based on NGS data, and Pedigree Finder (https://pedigree.db.naro.go.jp/) (Kajiya-Kanegae *et al.* 2022), which integrates pedigree and genomic information across breeding lines, are increasingly valuable for selecting breeding materials and designing markers. Most of the data in TASUKE+ pertains to genetic resources and registered cultivars, but its utility could be further enhanced by regularly incorporating information on recent breeding lines through breeder cooperation.

In response to rapid environmental and economic change, rice breeding now demands a wider range of traits. Advances in NGS technologies have made whole-genome sequencing (WGS) more accessible and cost-effective, enabling methods such as MutMap and QTL-seq for rapid allele identification (Abe *et al.* 2018). MutMap uses bulk WGS of selected individuals from a segregating population obtained by crossing a mutant with the original wild-type line and subsequently selfing, to infer genomic regions associated with the target phenotype based on biased SNP polymorphism patterns across the genome (Abe *et al.* 2012). QTL-seq applies a similar approach to populations from cultivar crosses with contrasting phenotypes (Takagi *et al.* 2013a). These methods bypass extensive marker development and fine mapping with NILs or CSSLs, accelerating QTL identification and MAS. A notable case is the salt-tolerant line ‘hst1’, derived from a ‘Hitomebore’ mutant population, which was developed as a countermeasure against salt damage caused by the tsunami following the Great East Japan Earthquake. Using MutMap, the causal SNP for salt tolerance was identified and a corresponding marker was developed within one year (Takagi *et al.* 2015). Subsequent backcrossing with ‘Hitomebore’ and application of MAS led to the release of the salt-tolerant cultivar ‘Kaijin’, which retained excellent quality comparable to that of ‘Hitomebore’, in 2015—just four years after the disaster. MutMap has also identified genes associated with specialized traits, such as low cesium uptake (Ishikawa *et al.* 2017). Its derivative method, MutMap-Gap, has demonstrated its utility by identifying the components of the rice blast true resistance gene *Pii* (Takagi *et al.* 2013b). QTL-seq has also yielded practical results, such as the identification of a novel field resistance QTL to rice blast from the cultivar ‘Nortai’, as well as QTLs for seedling vigor (Takagi *et al.* 2013a). These WGS-based techniques enable efficient QTL identification and MAS without complex experimental materials, and are expected to further advance marker-assisted breeding.

As the evaluation of quantitative traits requires consideration of numerous genetic factors, expectations are growing for more efficient selection strategies. One promising approach that has emerged in response to this need is genomic selection (GS). Unlike conventional QTL mapping, which uses markers to tag individual loci, GS integrates genome-wide markers into statistical models to estimate trait values, offering a powerful method for selecting complex traits. Furthermore, GS accounts for QTLs of both large and small effects, thereby capturing a greater proportion of the genetic variance and enabling more accurate selection for quantitative traits (Anilkumar *et al.* 2023, Bartholomé *et al.* 2022). The concept of GS was first proposed by Meuwissen *et al.* (2001) in the context of animal breeding. In rice breeding, GS research using actual breeding populations was initiated at the International Rice Research Institute (Spindel *et al.* 2015). As genotyping costs decline, GS models informed by GWAS-based insights into genetic architecture and population structure have shown potential to improve breeding efficiency. Furthermore, subsequent studies have suggested that enhancing the evaluation of relationships between environmental data and phenotypic traits may further improve the prediction accuracy of GS models (Spindel and McCouch 2016). In Japan, historical rice breeding trial data across the country are being systematically organized (Matsushita *et al.* 2024). By integrating these with genomic data of the constituent cultivars, high-accuracy prediction models can be developed. Taniguchi *et al.* (2025) conducted genomic prediction of heading date in rice by integrating 74,350 historical records with genomic data from 411 cultivars, along with environmental variables such as temperature and day length, and spatial effects related to regional field locations. Their model achieved a prediction accuracy of 5.11 days in RMSE and a correlation coefficient of 0.90, even for cultivars not included in the training dataset. Although the model did not fully account for epistatic interactions among genomic loci and thus leaves room for improvement, it successfully enabled location-specific prediction of heading date by incorporating environmental information. This level of accuracy supports its utility in narrowing down breeding materials suited to specific regional conditions. This framework is expected to be applicable to other traits such as grain yield. To further improve genomic prediction, rapid expansion of data infrastructure—including phenotypic and genomic information for newly developed cultivars and lines—is essential, along with the development of appropriate evaluation models that account for environmental influences and gene-gene interactions.

As breeding methodologies become increasingly sophisticated, there is a growing need to simultaneously investigate polymorphisms across many DNA markers. However, next-generation sequencers and advanced genotyping equipment required for such analyses are often too expensive for routine use in individual laboratories, and their operation and data analysis demand specialized expertise. To further promote the use of DNA markers and accelerate breeding, stronger collaboration is needed between institutions specializing in genomic analysis—whether public or private—and those focused on breeding. Enhanced information exchange between these organizations will likely lead to the more rapid and efficient development of breakthrough cultivars in the near future.

## Author Contribution Statement

KM contributed to writing the section titled “Accelerated Breeding Using MAS and Generation Advancement for Efficient Trait Stacking”. UY contributed to the investigation of the original sources of the markers mentioned in this review and to the compilation of the list. AG contributed to the completion of the remaining sections and to determining the overall structure of this review. All authors wrote the manuscript and approved the final version.

## Supplementary Material

Supplemental Table

## Figures and Tables

**Table 1.** Representative genes and QTLs utilized for marker-assisted selection in Japanese breeding programs

Gene/QTL name	Trait	Gene ID*/QTL	Chromosome	IRGSP-1.0 position (Mb)	Translated product	Representative developed cultivar(s)	Donor variety	Reference
*Pi9*	True blast resistance	LOC_Os06g17890–LOC_Os06g17900	6	10.38–10.39	NBS-LRR protein	Koshihikari Kanto BL1	75-1-127 (*indica*)	Qu *et al.* (2006)
*Pita-2*	True blast resistance	Os12g0285100	12	10.8	Atypical protein	Fu-Fu-Fu	PiNo.4	Takahashi *et al.* (2017)
*Pb1*	Field resistance to panicle blast	Os11g0598500 LOC_Os11g38580	11	22.86–22.87	NBS-LRR protein	Tachiharuka Niji-no-kirameki	Modan	Hayashi *et al.* (2010b)
*pi21*	Field resistance to leaf blast	Os04g0401000	4	19.84	Proline-rich protein	Tomohonami Fu-Fu-Fu	Sensho	Fukuoka *et al.* (2009)
*Pi39*	Field resistance to leaf blast	QTL	4	31.14–31.81	NA	Eminoaki Tachiharuka	Haonaihuan	Terashima *et al.* (2008)
*Stvb*	Resistance to rice stripe disease	Os11g0514000	11	18.39–18.40	Histidine kinase/HSP90-like ATPase superfamily	Koshihikari Kinchushi SBL1	Upland Rice Kanto72	Hayano-Saito and Hayashi (2020)
*Stvb-i*	Resistance to rice stripe disease	Os11g0514000	11	18.39–18.40	Histidine kinase/HSP90-like ATPase superfamily	Koshihikari Aichi SBL Tachiharuka Niji-no-kirameki	Modan	Hayano-Saito and Hayashi (2020)
*Stva*	Resistance to rice stripe disease	QTL	6	1.77–9.34	NA	Koshihikari Kinchushi SBL1	Upland Rice Kanto72	Maeda *et al.* (2006)
*bsr1*	Resistance to brown spot	QTL	11	23.69–25.44	NA	Mienoyume BSL	Tadukan	Matsumoto *et al.* (2021)
*Wx-mq*	Low amylose content (High eating quality)	Os06g0133000	6	1.77	Granule-bound starch synthase I	Himegonomi	Milky Queen	Sato *et al.* (2002)
*Wx1-1*	Low amylose content (High eating quality)	Os06g0133000	6	1.77	Granule-bound starch synthase I	Oborozuki Sansanmaru	Hokkai287	Ando *et al.* (2010)
*Wxa*	High amylose content (Suitability for noodle processing)	Os06g0133000	6	1.77	Granule-bound starch synthase I	Koshinokaori Ajianokaori	Surjamukhi	Yamanaka *et al.* (2004)
*sbe1*	Low pasting temperature	Os06g0726400	6	30.9	Starch branching enzyme 1	Yawakoimochi Yawaramaru	Hiderishirazu-D	Okamoto *et al.* (2013)
*Apq1* (*Sus3*)	High-temperature tolerance	Os07g0616800	7	25.43	Sucrose synthase III	Fu-Fu-Fu	Habataki	Takehara *et al.* (2018)
*osnramp5*	Low cadmium uptake	Os07g0257200	7	8.87–8.88	Manganese and cadmium transporter	Akitakomachi R	Koshihikari Kan No. 1	Ishikawa *et al.* (2012)
*hd1*	Early heading	Os06g0275000	6	9.34	Zinc finger protein	Milky Summer	Kasalath	Yano *et al.* (2000)
*DTH8*	Late heading	Os08g0174500	8	4.33	HAP3 CCAAT box-binding transcription factor	Milky Autumn	Kasalath	Wei *et al.* (2010)
*sd1*	Short culm (Lodging resistance)	Os01g0883800	1	38.38–38.39	Gibberellin 20 oxidase 2	Koshihikari Tsukuba SD1 Fu-Fu-Fu	Dee-geo-woo-gen	Sasaki *et al.* (2002)
*SCM1*	Lodging torelance	QTL	1	2.17–24.39/ 28.61–40.21	NA	Sakura Prince	Habataki	Ookawa *et al.* (2010)
*SCM3* (*MP3*)	Lodging torelance Panicle number	Os03g0706500	3	28.43	TCP family transcription factor	Sakura Prince	Chugoku117 Takanari	Yano *et al.* (2015) Takai *et al.* (2014)
*SCM4*	Lodging torelance	QTL	2	3.86–7.42	NA	Sakura Prince	Chugoku117	Yano *et al.* (2015)
*hst1*	salt-tolerant	Os06g0183100	6	4.14	B-type response regulator	Kaijin	Hitomebore mutant hst1 (EMS mutagenesis)	Takagi *et al.* (2015)

NA: not available *: Gene IDs are based on the IRGSP-1.0 annotation.
